# Supplementary figures and images for: Regional and temporal patterns of partisan polarization during the COVID-19 pandemic in the United States and Canada
Source: PLoS One. 2026 Apr 20;21(4):e0347327. doi: 10.1371/journal.pone.0347327 (PMC13095112; doi:10.1371/journal.pone.0347327)

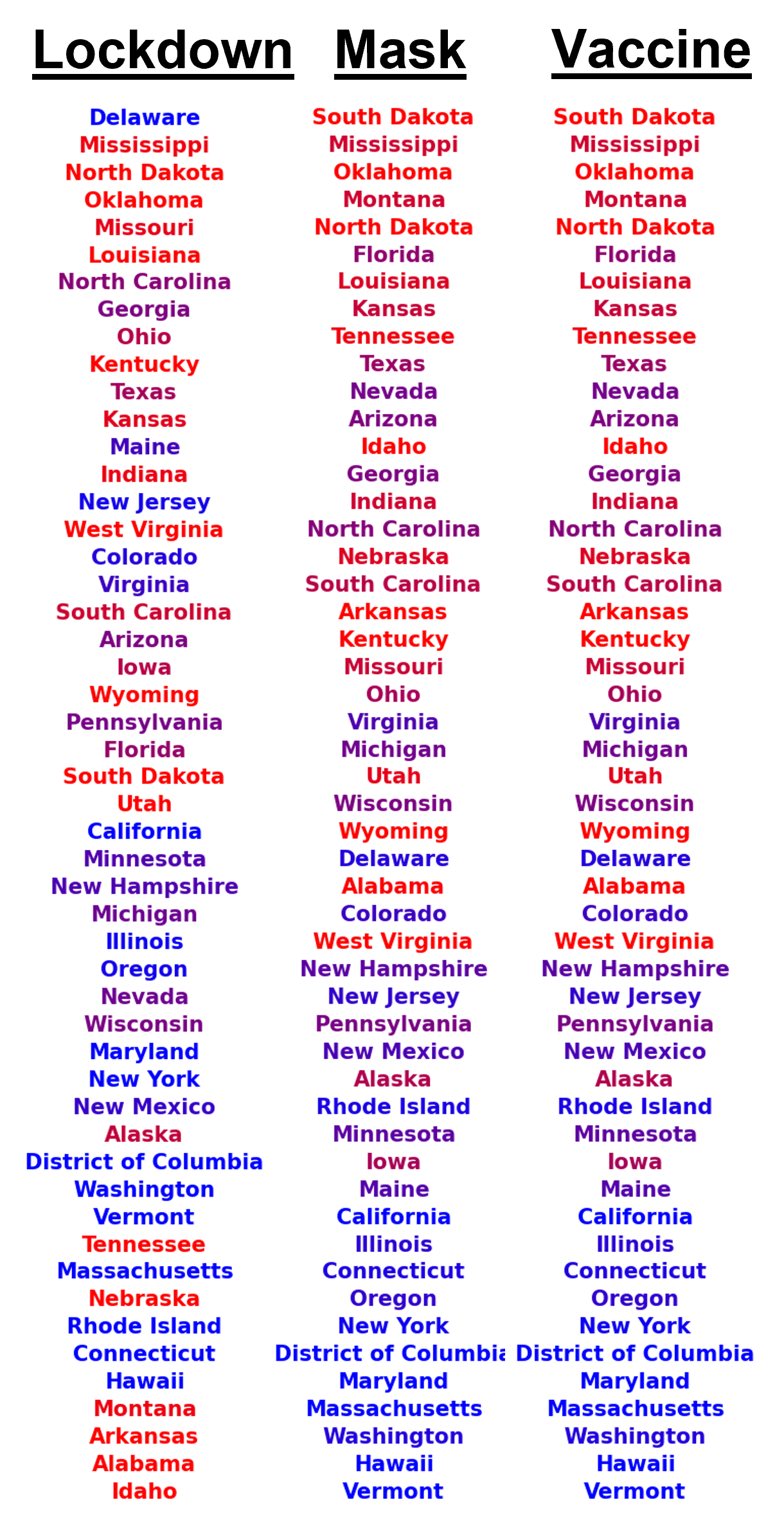

Supplement: S1 Fig — Ranking of 1 signifies the highest average weekly polarization between October 11, 2020 to January 3, 2021 (12 weeks). State names are colored based on the party ratio from the 2020 United States Presidential Election, where more blue means more users voted for the Democratic Party and more red means more users voted the Republican Party. We can see that red states are mostly ranked higher than blue states. (PNG) [file pone.0347327.s001.png]

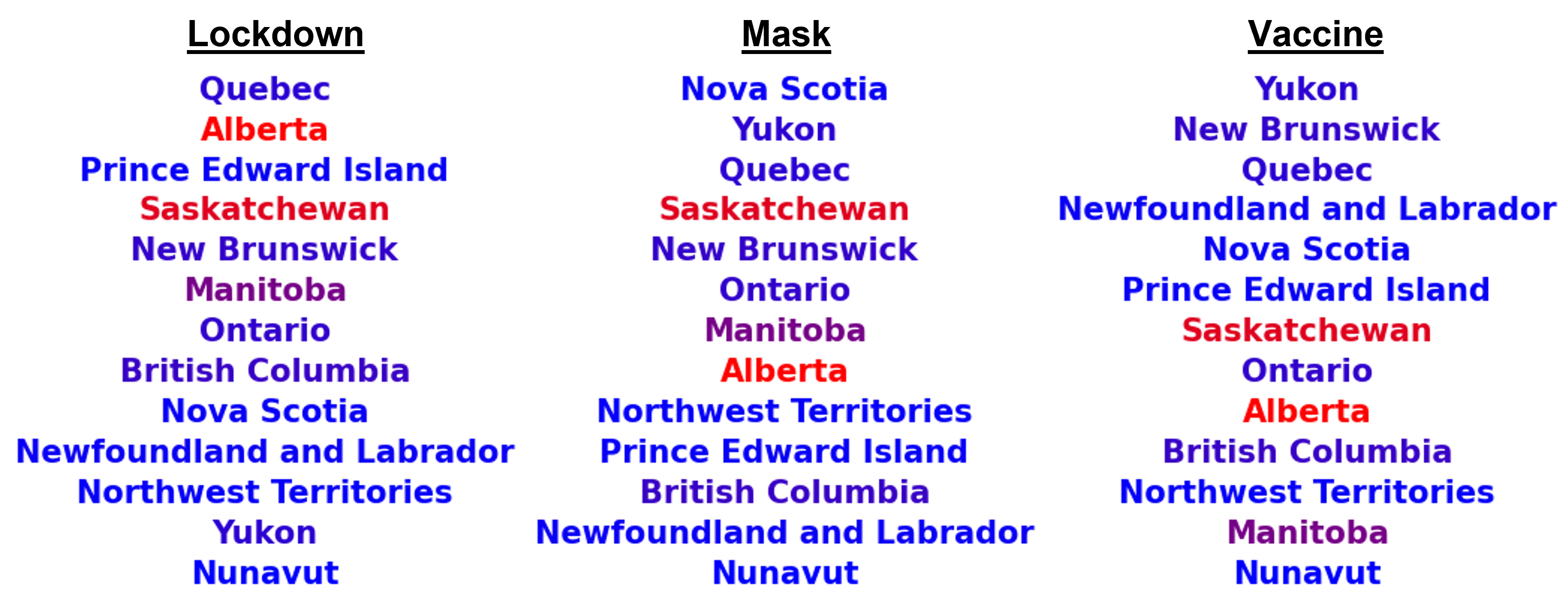

Supplement: S2 Fig — A ranking of 1 signifies the highest average weekly polarization between October 11, 2020 to January 3, 2021 (12 weeks). Province or territory names are colored based on the party ratio from Canada’s 2021 Federal Election, where more blue means more users from the liberal (left) party family (Liberal, New Democratic Party, Green), and more red means more users from the conservative (right) party family (Conservative, People’s Party). (PNG) [file pone.0347327.s002.png]

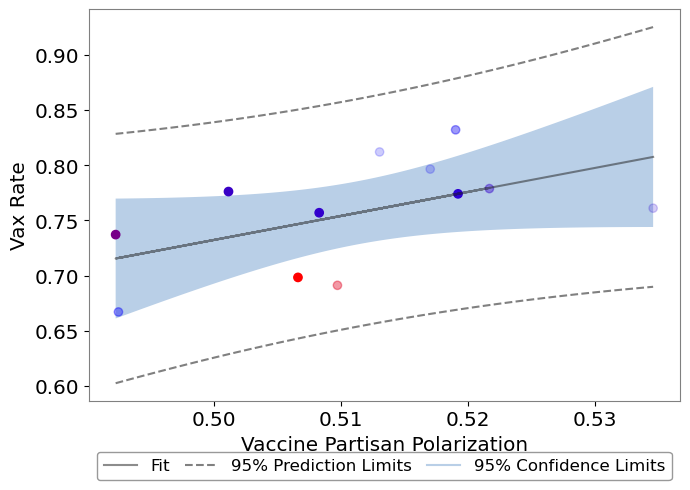

Supplement: S3 Fig — We remove Nunavut as an outlier because of its very small population. The correlation is 0.74 with CI = [0.31, 0.92] (n = 12, p = 0.004). The Vaccine Partisan Polarization for each province or territory is computed weekly and averaged over 12 weeks from October 11, 2020 to January 3, 2021. Official vaccination rates for different regions are obtained from Statistic Canada. The Vaccination Rate is also averaged weekly for the similar period of time a year into future to be after the vaccines were rolled out, i.e., October 11, 2021 to January 3, 2022. Color for the scatter plots is determined by the respective party ratio from the 2021 Canadian federal election. While we get strong positive correlation with vaccination rate, it is over a relatively low number of points. In Canada, vaccines were mandated, requiring vaccine passports to be served in public areas. We assume that vaccine partisan polarization increases, as people are not happy with being forced to be vaccinated, but most of the population still are vaccinated. However, with the few points, we do not have a definite conclusion for this result. (PNG) [file pone.0347327.s003.png]

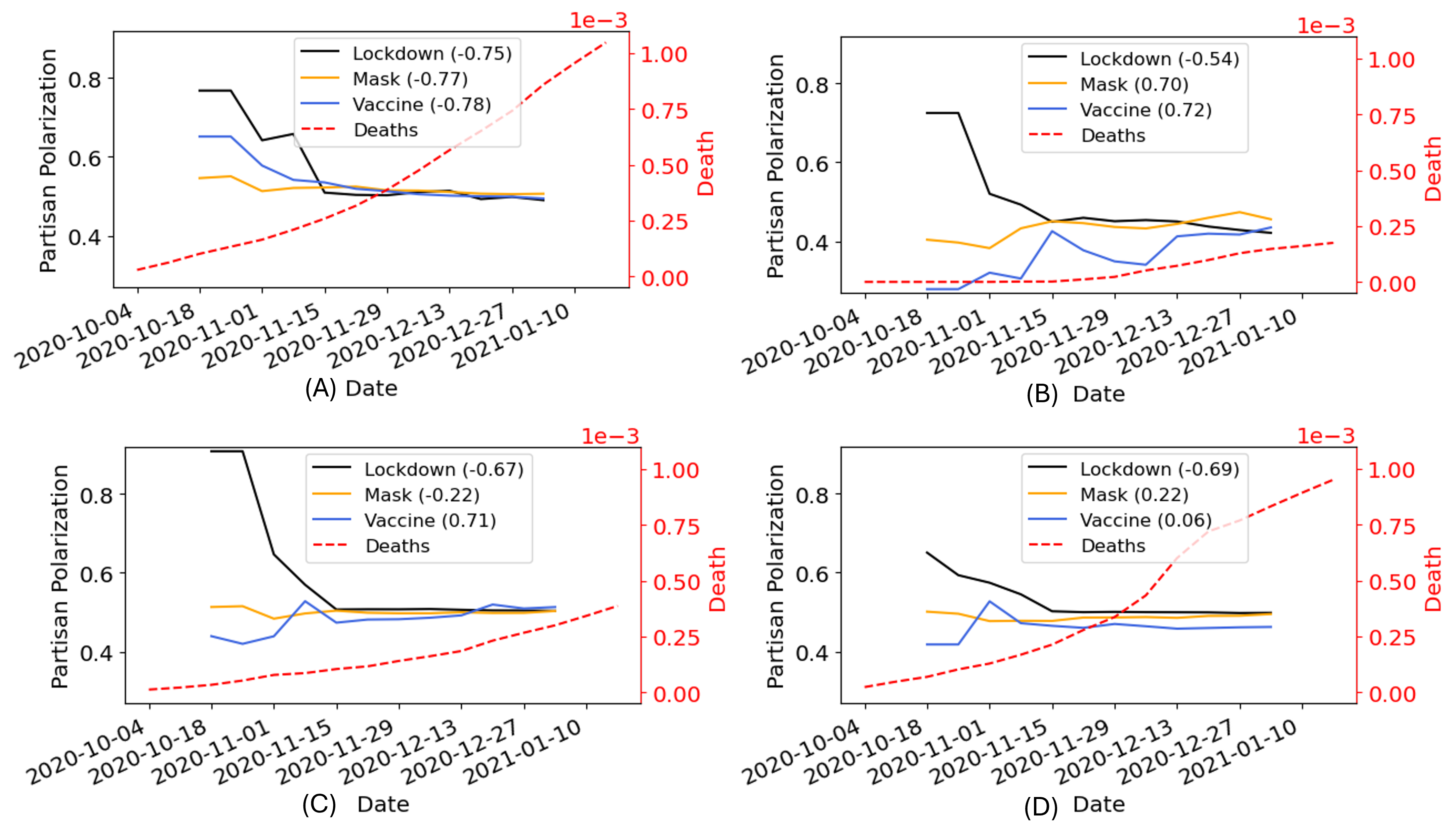

Supplement: S4 Fig — We present them for A: Highest ranked states overall (Mississippi). B: Lowest ranked state (Vermont). C: Highest ranked liberal state (Delaware). D: Lowest ranked conservative state (Iowa). We report the average death rate (red dotted line) per week and report the correlation between the topic-specific correlation with the death rate in the brackets in the legend. (PNG) [file pone.0347327.s004.png]

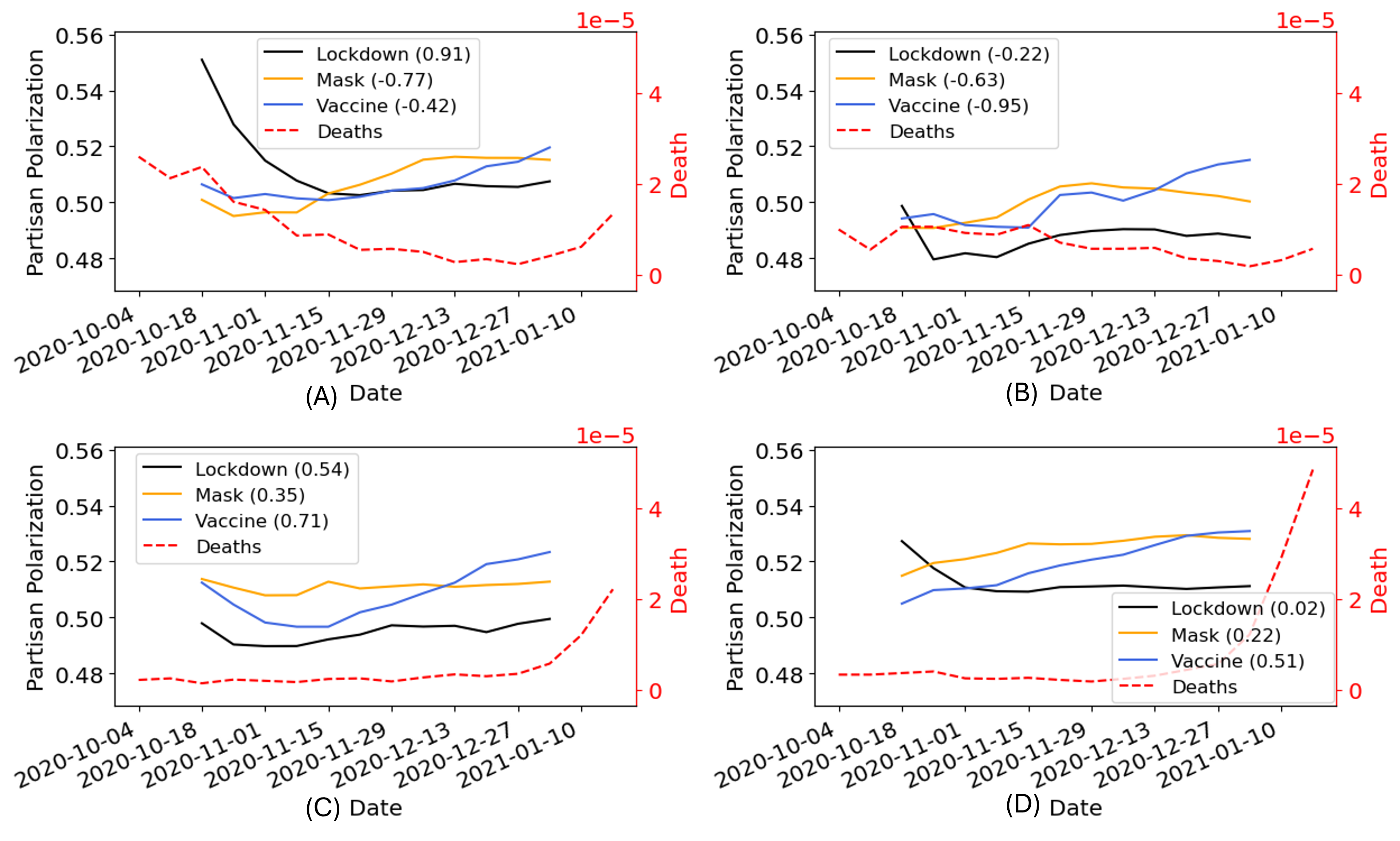

Supplement: S5 Fig — A: Alberta. B: British Columbia. C: Ontario. D: Quebec. We report the average death rate (red dotted line) per week and report the correlation between the topic-specific correlation with the death rate in the brackets in the legend. (PNG) [file pone.0347327.s005.png]

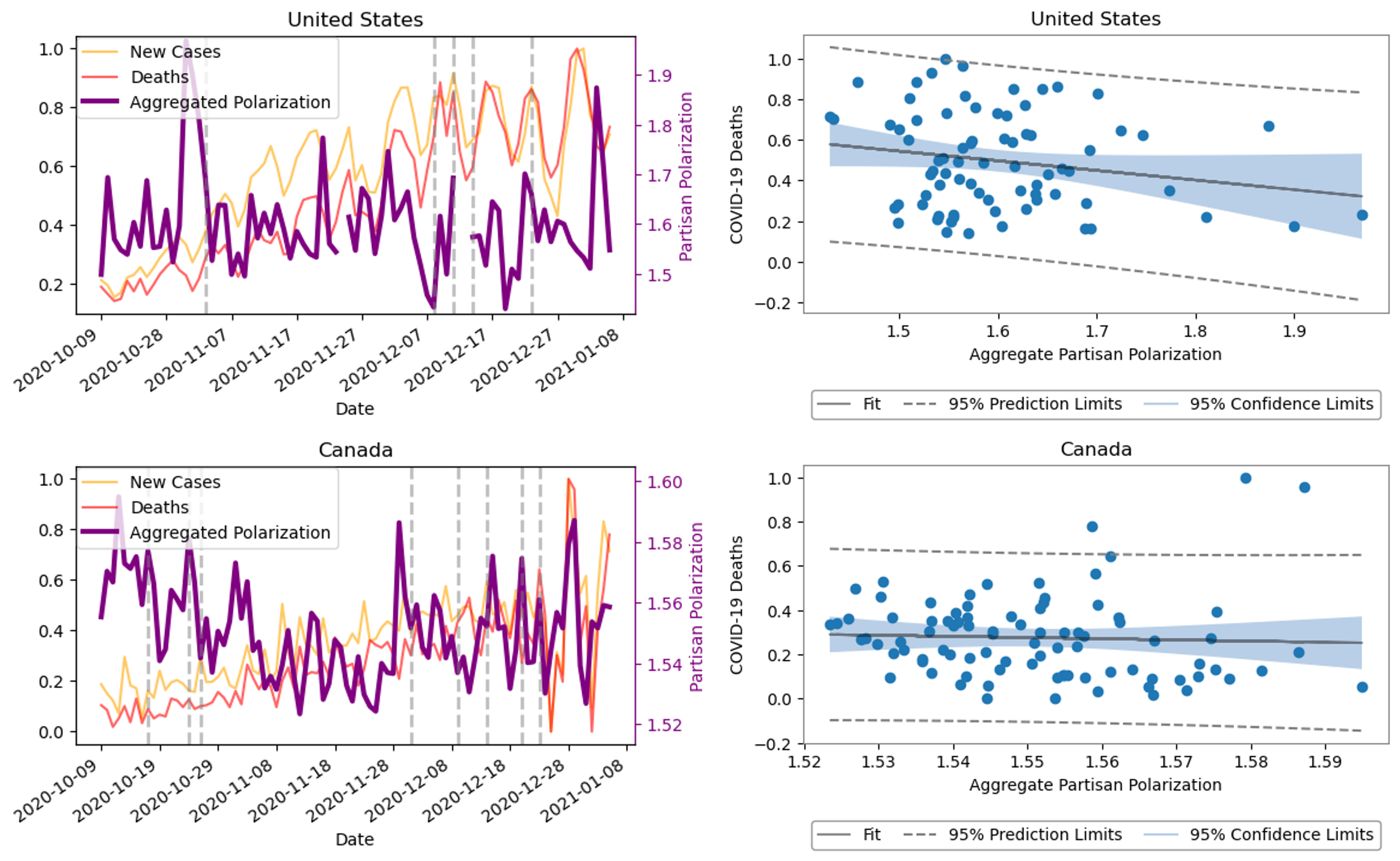

Supplement: S6 Fig — COVID-19 new cases and deaths for the United States and Canada. Here, we investigate the aggregated polarization over time for each country and how it relates to the reported number of New Cases and Deaths for COVID-19. To compute the daily aggregate polarization measure, we employ a weighted sum of each topic’s polarization, considering the percentage of each topic’s tweets within that day’s volume of COVID-19-related tweets. The correlation coefficient are −0.196 for the United States with CI = [−0.403,0.031] (n = 88, p = 0.090) and −0.044 for Canada with CI = [0.251,0.167] (n = 88, p = 0.681).We observe that polarization is not correlated with the severity of the pandemic, in both the United States and Canada. (PNG) [file pone.0347327.s006.png]
