# Supplementary material for: Regional and temporal patterns of partisan polarization during the COVID-19 pandemic in the United States and Canada
Source: PLoS One. 2026 Apr 20;21(4):e0347327. doi: 10.1371/journal.pone.0347327 (PMC13095112; doi:10.1371/journal.pone.0347327)
Supplement: S1 Table — Bolded means p < 0.001. Italicized means p < 0.01. Underline means p < 0.05. Background color of green or red signifies the positive or negative correlation for significant p-values only. (PDF) [file pone.0347327.s007.pdf]

Table S1. Correlation matrix between topic polarization and external Data in United States.

		Cases	Deaths	Conspiracy (Volume)	Stringency Index
Lockdown	Polarization	-0.020 CI=[-0.244,0.207] p=0.867	-0.070 CI=[-0.291,0.158] p=0.548	-0.146 CI=[-0.360,0.082] p=0.207	0.010 CI=[-0.216,0.235] p=0.931
	Volume	-0.196 CI=[-0.403,0.031] p=0.090	*-0.319 CI=[-0.508,-0.100] p=0.005*	**0.497 CI=[0.306,0.650] p=0.000**	-0.262 CI=[-0.460,-0.038] p=0.022
	% Volume	**-0.394 CI=[-0.569,-0.185] p=0.000**	**-0.436 CI=[-0.602,-0.233] p=0.000**	0.120 CI=[-0.109,0.336] p=0.303	*-0.356 CI=[-0.538,-0.142] p=0.002*
	Weighted Polarization	**-0.395 CI=[-0.570,-0.186] p=0.000**	**-0.442 CI=[-0.607,-0.240] p=0.000**	0.101 CI=[-0.128,0.319] p=0.387	*-0.354 CI=[-0.537,-0.140] p=0.002*
Mask	Polarization	-0.103 CI=[-0.321,0.125] p=0.376	-0.108 CI=[-0.325,0.121] p=0.355	-0.078 CI=[-0.298,0.150] p=0.502	-0.121 CI=[-0.337,0.107] p=0.298
	Volume	*-0.311 CI=[-0.501,-0.092] p=0.006*	*-0.378 CI=[-0.556,-0.167] p=0.001*	**0.500 CI=[0.310,0.652] p=0.000**	-0.288 CI=[-0.482,-0.067] p=0.012
	% Volume	**-0.541 CI=[-0.683,-0.360] p=0.000**	**-0.526 CI=[-0.672,-0.341] p=0.000**	-0.012 CI=[-0.237,0.214] p=0.915	**-0.452 CI=[-0.615,-0.252] p=0.000**
	Weighted Polarization	**-0.565 CI=[-0.701,-0.390] p=0.000**	**-0.547 CI=[-0.688,-0.367] p=0.000**	-0.020 CI=[-0.245,0.206] p=0.861	**-0.474 CI=[-0.632,-0.279] p=0.000**
Vaccine	Polarization	-0.246 CI=[-0.446,-0.021] p=0.033	-0.216 CI=[-0.421,0.010] p=0.061	-0.268 CI=[-0.466,-0.046] p=0.019	-0.133 CI=[-0.348,0.096] p=0.253
	Volume	*0.360 CI=[0.147,0.542] p=0.001*	*0.305 CI=[0.085,0.496] p=0.007*	*0.303 CI=[0.084,0.495] p=0.008*	0.196 CI=[-0.031,0.404] p=0.089
	% Volume	**0.666 CI=[0.518,0.775] p=0.000**	**0.682 CI=[0.539,0.786] p=0.000**	-0.068 CI=[-0.289,0.160] p=0.561	**0.573 CI=[0.400,0.707] p=0.000**
	Weighted Polarization	**0.633 CI=[0.475,0.751] p=0.000**	**0.655 CI=[0.505,0.767] p=0.000**	-0.115 CI=[-0.332,0.113] p=0.322	**0.576 CI=[0.403,0.710] p=0.000**
Aggregated	Sum	-0.184 CI=[-0.393,0.044] p=0.112	-0.196 CI=[-0.403,0.031] p=0.090	-0.247 CI=[-0.448,-0.023] p=0.031	-0.119 CI=[-0.335,0.110] p=0.307
	Weighted Sum	-0.217 CI=[-0.422,0.009] p=0.060	-0.198 CI=[-0.405,0.029] p=0.086	-0.193 CI=[-0.401,0.034] p=0.095	-0.091 CI=[-0.310,0.137] p=0.434

Bolded means $p < 0.001$. Italicized means $p < 0.01$. Underline means $p < 0.05$. Background color of green or red signifies the positive or negative correlation for significant p-values only.
