# Supplementary material for: Regional and temporal patterns of partisan polarization during the COVID-19 pandemic in the United States and Canada
Source: PLoS One. 2026 Apr 20;21(4):e0347327. doi: 10.1371/journal.pone.0347327 (PMC13095112; doi:10.1371/journal.pone.0347327)
Supplement: S2 Table — Bolded means p < 0.001. Italicized means p < 0.01. Underline means p < 0.05. Background color of green or red signifies the positive or negative correlation for significant p-values only. (PDF) [file pone.0347327.s008.pdf]

Table S2. Correlation matrix between topic polarization and external data in Canada.

		Cases	Deaths	Conspiracy (Volume)	Stringency Index
Lockdown	Polarization	***-0.338 CI=[-0.511,-0.138] p=0.001***	*-0.280 CI=[-0.462,-0.075] p=0.008*	0.190 CI=[-0.020,0.384] p=0.076	**-0.518 CI=[-0.657,-0.347] p=0.000**
	Volume	0.244 CI=[0.037,0.432] p=0.022	0.171 CI=[-0.040,0.367] p=0.112	-0.084 CI=[-0.288,0.128] p=0.437	0.259 CI=[0.052,0.444] p=0.015
	% Volume	**-0.368 CI=[-0.536,-0.172] p=0.000**	*-0.340 CI=[-0.513,-0.141] p=0.001*	0.149 CI=[-0.062,0.348] p=0.165	**-0.391 CI=[-0.555,-0.198] p=0.000**
	Weighted Polarization	**-0.402 CI=[-0.564,-0.210] p=0.000**	**-0.368 CI=[-0.536,-0.172] p=0.000**	0.172 CI=[-0.039,0.368] p=0.110	**-0.443 CI=[-0.597,-0.258] p=0.000**
Mask	Polarization	0.117 CI=[-0.095,0.318] p=0.279	0.102 CI=[-0.110,0.305] p=0.345	-0.132 CI=[-0.332,0.080] p=0.221	0.011 CI=[-0.198,0.220] p=0.916
	Volume	-0.202 CI=[-0.395,0.008] p=0.059	*-0.306 CI=[-0.485,-0.104] p=0.004*	**0.462 CI=[0.280,0.612] p=0.000**	-0.267 CI=[-0.452,-0.061] p=0.012
	% Volume	**-0.688 CI=[-0.784,-0.559] p=0.000**	**-0.689 CI=[-0.785,-0.561] p=0.000**	**0.615 CI=[0.465,0.730] p=0.000**	**-0.804 CI=[-0.867,-0.715] p=0.000**
	Weighted Polarization	**-0.687 CI=[-0.783,-0.557] p=0.000**	**-0.688 CI=[-0.784,-0.559] p=0.000**	**0.610 CI=[0.459,0.726] p=0.000**	**-0.804 CI=[-0.867,-0.715] p=0.000**
Vaccine	Polarization	0.271 CI=[0.066,0.455] p=0.011	*0.361 CI=[0.164,0.530] p=0.001*	-0.202 CI=[-0.395,0.007] p=0.059	0.254 CI=[0.047,0.440] p=0.017
	Volume	**0.718 CI=[0.599,0.806] p=0.000**	**0.658 CI=[0.520,0.762] p=0.000**	**-0.364 CI=[-0.533,-0.168] p=0.000**	**0.711 CI=[0.590,0.801] p=0.000**
	% Volume	**0.683 CI=[0.553,0.781] p=0.000**	**0.672 CI=[0.538,0.773] p=0.000**	**-0.532 CI=[-0.667,-0.363] p=0.000**	**0.781 CI=[0.684,0.851] p=0.000**
	Weighted Polarization	**0.687 CI=[0.558,0.784] p=0.000**	**0.678 CI=[0.546,0.777] p=0.000**	**-0.533 CI=[-0.668,-0.364] p=0.000**	**0.782 CI=[0.684,0.852] p=0.000**
Aggregated	Sum	-0.121 CI=[-0.322,0.091] p=0.261	-0.044 CI=[-0.251,0.167] p=0.681	0.023 CI=[-0.187,0.231] p=0.831	*-0.315 CI=[-0.492,-0.113] p=0.003*
	Weighted Sum	-0.062 CI=[-0.268,0.149] p=0.566	0.027 CI=[-0.183,0.236] p=0.799	-0.032 CI=[-0.240,0.178] p=0.764	-0.256 CI=[-0.442,-0.049] p=0.016

Bolded means $p < 0.001$. Italicized means $p < 0.01$. Underline means $p < 0.05$. Background color of green or red signifies the positive or negative correlation for significant p-values only.
