# Supplementary material for: Regional and temporal patterns of partisan polarization during the COVID-19 pandemic in the United States and Canada
Source: PLoS One. 2026 Apr 20;21(4):e0347327. doi: 10.1371/journal.pone.0347327 (PMC13095112; doi:10.1371/journal.pone.0347327)
Supplement: Attachment — Submitted filename: Response to Reviewers.pdf [file pone.0347327.s010.pdf]

Dear Reviewers,

We sincerely thank you and the reviewers for the thoughtful and constructive feedback on our manuscript “Regional and temporal patterns of partisan polarization during the COVID-19 pandemic in the United States and Canada” (ID: PONE-D-25-11475). We truly appreciate the time and expertise invested in evaluating our work. We have carefully revised the manuscript in response, improving the positioning within existing literature, refining methodological transparency, regenerating figures, and expanding limitations to address representativeness, temporal, and methodological concerns. We also revised the overall framing to ensure informative, fact-based rather than evaluative language.

Below we provide a detailed point-by-point response to the editor’s synthesis and to the individual reviewer comments. Reviewer comments are paraphrased in bold italics, followed by our responses.

Editor’s Comments:

Editor Comment	Response	Manuscript Change
1. Introduction requires better positioning in literature, especially regarding LLMs and the political/temporal context.	Expanded introduction to synthesize prior literature on both classic and embedding-based approaches; contextualized study within both U.S./Canadian temporal frames.	Added balanced citations for both countries and for embedding-based work; revised narrative to reflect both temporal and political context.
2. Methodological rationale unclear; need comparison to network-based approaches and formal operational definitions.	Added explicit rationale for text similarity, compared network-based methods, and provided definitions for key constructs.	Added side-by-side benefits/drawbacks of text vs. network-based approaches. Defined constructs such as conspiracy and sentiment in Methods.
3. Figures and tables are low quality and overloaded.	All figures regenerated at high resolution and simplified; tables and figures annotated and renamed for clarity.	Updated visuals throughout the manuscript; legends clarified; missing and mislabeled figures/tables corrected.

4. Figure 2, 3, 7, 8 copyrights	Figures 2, 3, 7, and 8 were fully generated using Python's Bokeh library and are original to the authors.	No change
5. Limitations do not justify temporal differences or address issues noted by reviewers.	Expanded limitations to discuss temporal mismatches, data representativeness, location/bot issues, and keyword bias.	Added dedicated paragraphs on temporal limitations, geolocation/bot bias, and cross-national differences in keyword strategy.
6. Language too evaluative; needs more concrete, fact-based description.	Thoroughly revised for informative and concrete language; fact-based statements favored over evaluative descriptors.	Replaced vague or broad phrasing throughout; fixed capitalization and party labeling inconsistencies.

Reviewer 1 Comments:

Reviewer 1 Comment	Response	Manuscript Change
1. Reliance on text similarity rather than stance or network structure; needs comparison to network-based alternatives.	Added explicit comparison to network-based methods and justified text-embedding approach due to access and scalability.	Included citations (Conover et al., Barberá, Stella) and comparative discussion in introduction and methods.
2. Novelty claim overstates impact; should better position against prior LLM applications (e.g., He et al.).	Expanded literature review with recent, directly relevant text embedding studies; clarified our specific contribution.	Added new citations, reframed narrative to clarify innovation as fine-grained region/event tracking.

3. Data filtering keywords terminology unclear (line 108).	Renamed “topic-specific keywords” as “political” and defined separately from “topic-specific keywords”.	Updated terminology and clarifications in the Methods section.
4. Temporal difference between tweet collection and vaccination data lacks justification.	Now described in Methods/Limitations as exploratory, predictive—not causal—analysis; discussed implications.	Added rationale and expanded relevant paragraphs clarifying timing and interpretation.
5. RoBERTa fine-tuning and evaluation procedures unclear (lines 149-152, Table 1 clarification).	Clarified that RoBERTa was fine-tuned on all hashtag-annotated tweets and evaluated on manually validated sample; defined “five runs” in Table 1.	Expanded methods and added explicit details about annotation, fine-tuning, and sampling strategy.
6. Geolocation method unclear (line 159).	Clarified use of Open Street Map and ArcGIS API for location, not pre-trained NER.	Methods revised for explicit geo-processing description.
7. Figures missing captions, poor quality, redundant naming, missing subfigures.	Regenerated all figures, added unique captions and axis labels; named subfigures consistently; included any omitted items.	Updated all figures and legends.
8. Table numbering and supplementary materials inconsistencies.	Corrected numbering and labeling throughout manuscript.	Re-numbered and cross-referenced tables for clarity.
9. User–voter record matching method underexplained.	Expanded description to include possible errors (location, names, bots).	Added comprehensive details in Methods and Limitations section.

Reviewer 2 Comments:

Reviewer 2 Comment	Response	Manuscript Change
1. Abstract underemphasizes Canada and does not mention representativeness issue.	Explicitly noted that the polarization index reflects engaged Twitter users, and expanded Canadian context and findings.	Abstract edited to address both concerns.
2. U.S. dominance in introduction; socio-demographic citation too narrow.	Brought more Canadian context and replaced elite-focused citation with broader, population-level studies.	Updated introduction with balanced coverage and accurate citations.
3. Temporal mismatch between discourse and vaccination rates.	Clarified that analysis is exploratory and reflects predictive, not causal, associations.	Methods and limitations revised to explain the timing issue.
4. US/Canada keyword lists differ, biasing topical coverage; explanation lacking.	Explicitly acknowledged and justified keyword strategy asymmetry and discussed impact on results.	Expanded methods and limitations with evaluation of keyword bias.
5. Conspiracy and sentiment classification unclear, sarcasm/satire not detected.	Clarified conspiracy classification method (pro/con), and acknowledged semantic similarity metric may miss sarcasm/satire.	Methods and limitations updated with details and acknowledgments of limitations.
6. Tweet activity thresholds not explained.	Included justification and sensitivity analysis for tweet threshold selection.	Methods section now clarifies rationale and tested thresholds.
7. CDC vaccination normalization process not described for U.S.	Clarified that vaccination data for U.S. and Canada normalized by population across all regions.	Methods updated for consistency.
8. Broad terms used (e.g., “comprehensive approach”) without definition.	Revised text to remove vague or broad descriptors; stated definitions upfront.	Edited introduction and methods for clearer phrasing.

9. Party capitalization inconsistency; minor style issues.	Corrected all party/capitalization errors.	Consistent party naming throughout.
10. Location errors, VPNs, bot influence, and representativeness not discussed in limitations.	Added detailed acknowledgments of geolocation inaccuracies and bot/VPN influence in limitations.	Limitations section expanded and restructured.

We believe these major revisions have greatly improved clarity, rigor and transparency of the manuscript while addressing every concern from both reviewers. We respectfully submit the revised version for consideration and remain grateful for your guidance in strengthening this work.

With sincere thanks and best regards,

Zachary Yang
On behalf of all co-authors
