# Supplementary material for: Regional and temporal patterns of partisan polarization during the COVID-19 pandemic in the United States and Canada
Source: PLoS One. 2026 Apr 20;21(4):e0347327. doi: 10.1371/journal.pone.0347327 (PMC13095112; doi:10.1371/journal.pone.0347327)
Supplement: Attachment — Submitted filename: Response to Reviewers_v3.pdf [file pone.0347327.s011.pdf]

Dear Reviewers,

We sincerely thank you and the reviewers for the thoughtful and constructive feedback on our manuscript “Regional and temporal patterns of partisan polarization during the COVID-19 pandemic in the United States and Canada” (ID: PONE-D-25-11475).

We truly appreciate the time, care, and expertise invested in evaluating our work. We have carefully reviewed all comments and have revised the manuscript where appropriate to improve clarity, rigor, and transparency.

Below, we provide a detailed point-by-point response.

Reviewer 1 Comments:

Reviewer 1 Comment	Response	Manuscript Change
This text appears twice: "tion rates are for those who obtained at least two doses."	Thank you for catching this duplication.	The duplicated text (Lines 141–142) has been removed.
This sentence seems incomplete:<br><br>vent-triggered average polarization for identified events listed in Table 9 and Table 10 respectively.	We appreciate this observation. The sentence in the caption for Figure 14 was indeed fragmented.	The caption has been revised to read:<br><br>Event-triggered average polarization for identified events are listed in Table 9 and Table 10 respectively.

Reviewer 3 Comments:

We appreciate the detailed feedback. Several comments appear to reference elements (e.g., specific claims, tables, figures, or section numbers) that are not present in our submitted manuscript. To ensure clarity, we respond carefully below and clarify where misunderstandings may have occurred

Reviewer 3 Comment	Response	Manuscript Change
The authors state that the proposed approach “clearly outperforms existing methods and demonstrates robust generalizability”.However, the evidence presented in Table 3 and Figure 4 shows performance improvements that are relatively modest and, in some cases, overlapping within variance ranges. For example, the reported improvement over the baseline method in Table 3 is small on Dataset B, and no statistical significance testing is reported to confirm whether this difference is meaningful. I recommend either (i) tempering claims of “clear” or “robust” superiority, or (ii) adding appropriate statistical tests to support these statements.	We carefully reviewed the manuscript but were unable to locate the quoted statement (“clearly outperforms existing methods and demonstrates robust generalizability”). Our manuscript does not make claims of superiority over existing methods in this manner.<br><br>Regarding the referenced materials:<br><br>● **Table 3** presents party affiliation classification results. No external baseline method is compared. We report the mean F1-score and standard deviation across 5 independent runs (random seeds).<br><br>● **Figure 4** presents the approximation error and runtime tradeoff for polarization	The captions of Tables 3 and 4 now explicitly state that results are averaged over 5 random seeds with reported standard deviation.

	computation. The figure illustrates that approximation error remains small (approximately 0.001 absolute error) while computational savings increase substantially as data size grows.<br><br>To improve clarity, we have now explicitly stated in the caption and text that classification results are averaged over multiple random seeds.	
Statistical methodology is insufficiently described; absence of statistical testing; unclear whether results are averaged.	We clarify the following:<br><br>● **Table 2** reports correlation statistics with sample size (n), p-values, and 95% confidence intervals (e.g., 0.98 (n=52, p=9.27e-35, CI=[0.96, 0.99])).<br><br>● **Tables 3 and 4** report mean F1-scores and standard deviations across 5 random seeds.<br><br>● All classification results are averaged over	Tables 3 and 4 now specify averaging over 5 random seeds and report standard deviation explicitly.

	multiple runs.<br><br>To avoid ambiguity, we have explicitly added this information to the relevant captions..	
Figure 5 error bars unclear.	Figure 5 does not contain error bars. We believe this comment may refer to Figure 4. To improve clarity, we have now explicitly stated in both the main text and caption that the results shown are averaged over 10 independent runs and that the plotted variation represents standard deviation.	Figure 4 caption updated to clarify averaging over 10 independent runs and reporting of standard deviation.
Data availability	Due to platform policy restrictions, the full Twitter dataset cannot be publicly redistributed. However:<br><br>● All Tweet IDs used in the analysis will be publicly released.<br><br>● All trained language models will be hosted publicly on Hugging Face.<br><br>● Analysis code and preprocessing scripts will be released in a public	

	repository upon publication.	
Methodological details insufficient (Section 3.3).	The submitted manuscript does not contain a Section 3.3 titled “Model Configuration / Experimental Setup.” We believe this comment may refer to a different manuscript. However, we have re-reviewed our Methods section to ensure clarity and completeness.	No change.
Claims in Related Work about prior methods being “limited” or “ineffective.”	We carefully searched the manuscript and do not use the terms “limited” or “ineffective”..	No change.
Figure 2 caption should better describe the experimental pipeline.	Figure 2 does not represent an experimental pipeline.	No change.
Table 4 best-performing values highlighted without criteria.	Table 4 does not contain highlighted values. We believe this comment may refer to a different manuscript.	No change.
Grammatical and typographical errors.	The entire manuscript has been carefully proofread and reviewed using a grammar and style checker. Minor grammatical and tense inconsistencies were corrected throughout.	Minor changes

We believe these revisions have improved clarity, transparency, and precision while addressing all substantive concerns raised by the reviewers.

We are grateful for the reviewers’ time and thoughtful feedback, which has strengthened the manuscript. We respectfully submit the revised version for your consideration.

With sincere thanks and best regards,

Zachary Yang
On behalf of all co-authors
